# Bio-Control of *Salmonella* Enteritidis in Foods Using Bacteriophages

**DOI:** 10.3390/v7082847

**Published:** 2015-08-24

**Authors:** Hongduo Bao, Pengyu Zhang, Hui Zhang, Yan Zhou, Lili Zhang, Ran Wang

**Affiliations:** 1 Institute of Food Safety, Jiangsu Academy of Agricultural Sciences, State Key Laboratory Cultivation Base of MOST- Jiangsu Key Laboratory of Food Quality and Safety, Nanjing 210014, China; E-Mails: baohongduo@163.com (H.B.); zh851200@163.com (H.Z.); zhou.yan.77@hotmail.com (Y.Z.); lilizhangnj@163.com (L.Z.); 2 Ginling College, Nanjing Normal University, Nanjing 210097, China; E-Mail: panpan.jump@163.com

**Keywords:** *Salmonella* Enteritidis (SE), phages, phage cocktail, food, bio-control

## Abstract

Two lytic phages, vB_SenM-PA13076 (PA13076) and vB_SenM-PC2184 (PC2184), were isolated from chicken sewage and characterized with host strains *Salmonella* Enteritidis (SE) ATCC13076 and CVCC2184, respectively. Transmission electron microscopy revealed that they belonged to the family *Myoviridae*. The lytic abilities of these two phages in liquid culture showed 10^4^ multiplicity of infection (MOI) was the best in inhibiting bacteria, with PC2184 exhibiting more activity than PA13076. The two phages exhibited broad host range within the genus *Salmonella*. Phage PA13076 and PC2184 had a lytic effect on 222 (71.4%) and 298 (95.8%) of the 311 epidemic *Salmonella* isolates, respectively. We tested the effectiveness of phage PA13076 and PC2184 as well as a cocktail combination of both in three different foods (chicken breast, pasteurized whole milk and Chinese cabbage) contaminated with SE. Samples were spiked with 1 × 10^4^ CFU individual SE or a mixture of strains (ATCC13076 and CVCC2184), then treated with 1 × 10^8^ PFU individual phage or a two phage cocktail, and incubated at 4 °C or 25 °C for 5 h. In general, the inhibitory effect of phage and phage cocktail was better at 4 °C than that at 25 °C, whereas the opposite result was observed in Chinese cabbage, and phage cocktail was better than either single phage. A significant reduction in bacterial numbers (1.5–4 log CFU/sample, *p* < 0.05) was observed in all tested foods. The two phages on the three food samples were relatively stable, especially at 4 °C, with the phages exhibiting the greatest stability in milk. Our research shows that our phages have potential effectiveness as a bio-control agent of *Salmonella* in foods.

## 1. Introduction

*Salmonella* is a gram-negative bacterium that is one of the principal causes of food-borne diseases. Presently, over 2500 serotypes of *Salmonella* are known [1], and the most common worldwide is *Salmonella* Enteritidis (SE) [2]. SE is a food-borne pathogen that is a significant food safety concern globally. Since the period 1996–1999, the recorded incidence of human SE infection in the Foodborne Diseases Active Surveillance Network (FoodNet) has increased by 44% [3]. In Canada, SE was detected in various commodities, most frequently in chicken (with PT13, PT8 and PT13a predominating) [4]. In China, Ke’s study of 1764 clinical *Salmonella enterica* isolates in Guangdong province showed that ~15% of isolates were SE, which was the primary cause of salmonellosis in adults [5]. Despite improved preventive and control strategies in chicken commercial flocks and in the food industry, SE infection still poses a constant problem [6,7,8,9]. Moreover, with the misuse of antimicrobials in many farms including disease treatment and growth promotion in domestic livestock, many SE are resistant to several antimicrobial agents.

New environmentally-friendly intervention strategies are needed to treat microbial infection. In recent years, applications of bacteriophages to control bacterial pathogens have received new interest [10]. They have also been identified as a prospective alternative bio-control method for infections and contaminations by antimicrobial resistant pathogens [11]. Bacteriophages, or phages, are abundant in the environment, with an estimated ratio of 10:1 to their bacterial hosts. In many experimental studies, phages have been used to control *Salmonella* contamination in a variety of foods. Goode *et al.* [12] used phages to well-control SE on chicken skin that had been inoculated with commercially relevant numbers of bacteria (*i.e*., 1 log CFU/cm^2^) . In Leverentz’s study [13], *Salmonella*-specific phage could reduce *Salmonella* numbers in experimentally contaminated fresh-cut melons and apples stored at various temperatures. They also found that a phage mixture was better in reducing *Salmonella* populations than chemical sanitizers on honeydew melon slices [13]. The *Salmonella* phage (Felix-O1), which has a broad host range within the genus *Salmonella*, demonstrated an approximately about 2 log units reduction in *Salmonella* Typhimurium DT104 inoculated on chicken frankfurters [14]. In addition, phages had successfully controlled the growth of other important pathogens such as *Listeria monocytogenes* [15,16], *Escherichia coli* O157:H7 [17,18], and *Shigella* [19].

Our study involved the isolation from chicken excretion sewage of two new lytic SE phages using SE ATCC13076 and CVCC2184 as hosts. We determined their host ranges and lytic activity against hosts *in vitro*. The major aim of this study was to evaluate the potential of the two individual phages, or a mixture of the two (cocktail), to control SE contamination in three kinds of foods.

## 2. Materials and Methods

### 2.1. Salmonella Cultures, Media and Growth Conditions

A total of 311 epidemic *Salmonella* spp. strains were used in this study. Some of the strains were kindly donated by Guoxiang Cao (Chinese Academy of Agricultural Sciences, Yangzhou, China), Guoqiang Zhu (Yangzhou University, Yangzhou, China), Yuqing Liu (Shandong Academy of Agricultural Sciences, Jinan, China), Yanbing Zeng (Jiangxi Academy of Agricultural Sciences, Nanchang, China) and Jiansen Gong (Poultry institute, Chinese Academy of Agricultural Sciences, Yangzhou, China). The others were isolated between 2010 and 2015 from chicken farms and foods by our laboratory and stored in our lab. Of these, *S*. Enteritidis (SE) ATCC13076 and CVCC2184 were used to isolate bacteriophages. They were grown at 37 °C in Luria-Bertani broth (LB, Beijing land bridge technology Co., LTD, Beijing, China) or LB supplemented with 1.5% agar.

### 2.2. Isolation and Purification of Lytic Salmonella Phages

Fifty chicken excretion sewage samples were collected from 10 chicken farms in Jiangsu Province, China. Then these samples were used to isolate *Salmonella* phage as previously described [20]. Phages were propagated on SE ATCC13076 and CVCC2184 using LB (0.6% agar) soft agar overlays [21]. Incubating the double-layer agar plates for a longer period of time (3–4 days), the host cells that survive in the middle of the plaque were surveyed for whether they are resistant strains or lysogens.

To prepare high titers of phage, crude phage lysate propagating using a single plaque with its hosts was filtered a 0.22 μm filter, then NaCl (0.5 M, final concentration) and polyethylene glycol (PEG 8000) (10%, final concentration) (Amersco, Solon, OH, USA) were added to the supernatant and incubated overnight at 0 °C. Finally, phage particles were precipitated by centrifugation (10,000× *g*, 15 min, 4 °C), and resuspended in SM buffer (5.8 g/L of NaCl, 2.0 g/L of MgSO_4_, 50 mL/L of 1 M Tris, pH 7.5, 5 mL/L of presterilized 2% gelatin). The final concentration was 1.0 × 10^10^ PFU/mL.

### 2.3. Morphology of the Isolated Phages

Freshly-purified phage suspended pellets in SM buffer were used in this experiment. A small drop of phage was loaded onto a carbon-coated copper mesh grid and excess phage suspension was removed with filter paper. Negative staining of phages with 1% (*w*/*v*) phosphotungstic acid and phage images were obtained by a Transmission Electron Microscope (TEM) (H-7650, Hitachi High-Technologies Corporation, Tokyo, Japan) at an acceleration voltage of 80 kV.

### 2.4. Thermal and pH Stability

For thermal-stability testing, tubes with phages were kept in a water bath ranging from 30 to 90 °C for 30 min or 60 min. For pH-stability testing, samples of phages were mixed in a series of tubes containing buffer peptone water (BPW) of different pH (adjusted using NaOH or HCl) and incubated for 2 h at 37 °C. Bacteriophage titers were all determined using the double-layer agar plate method.

### 2.5. In Vitro Experiment of Phage Mediated Lysis

Overnight cultures of *Salmonella* ATCC13076 and CVCC2184 were diluted to ~10^4^ CFU/mL in fresh medium. A single phage stock (PA13076 or PC2184) was added to give three multiplicity of infection (MOI) of 10^2^, 10^3^ and 10^4^, respectively. The mixtures were then incubated at 37 °C for 5 h with gentle shaking. Phage-free culture (containing only bacteria) and bacteria-free culture (containing only phage) were also included as controls. Bacterial counts were determined at 0, 0.5, 1, 2, 3, 4 and 5 h.

### 2.6. Host Ranges of Phage

Three hundred eleven strains of epidemic *Salmonella* spp. were used in this study. Host ranges of phage were determined by spot test. The method was spotting 10 μL of phage preparation (~10^8^ PFU/mL) on lawn cultures of the bacteria strains. Plaque formation was monitored after incubation at 37 °C for 24 h.

### 2.7. Food Sample Preparation

Three different kinds of foods were purchased at local supermarkets and included: chicken breast, pasteurized whole milk and Chinese cabbage. All food samples were preliminarily analyzed to check for the possible natural contamination by *Salmonella*, according to the standard procedures. Samples of chicken breast were sliced aseptically into 2 cm × 2 cm squares (about 1 g), and placed into Petri dishes. Pasteurized whole milk samples were divided into 1 mL/sample in a biosafety cabinet. Chinese cabbage was washed with water, followed by washing with sterile water and 75% ethanol, prior to its use in the assays. The sanitized cabbage samples were then sliced into 2 cm × 2 cm squares, and placed in Petri dishes.

### 2.8. Individual Phage Treatment of Their Respective Hosts

SE ATCC13076 and CVCC2184 were grown individually in LB at 37 °C overnight. Phages PA13076 and PC2184 were used individually to challenge their appropriate hosts which were added to the three kinds of foods. Experiments were conducted at two temperatures (4 or 25 °C) to represent refrigeration and room temperatures. For each sample, 25 μL of diluted host strains (4 × 10^5^ CFU/mL) were carefully pipetted onto the surface of the meat and cabbage or into the milk samples and allowed to attach for 15 min at room temperature in a biosafety cabinet. This was followed by adding 25 μL of diluted phages (4 × 10^9^ PFU/mL) per sample. For the controls, the same volume of SM buffer was used instead of the phage suspension. All samples were performed in triplicate. Bacteria and its phage were monitored by viability counting on LB plates after 0, 1, 2, 3, 4 and 5 h of phage treatment. To quantify *Salmonella*, each chicken breast piece and Chinese cabbage was homogenized in 5 mL PBS. *Salmonella* were detected directly in milk samples. *Salmonella* were counted by pouring larger aliquots (1 mL) of diluted or undiluted of milk sample or the homogenates with molten LB agar on 90-mm plates. Simultaneously, the concentration of phage was determined at each of the monitored times. Phage counts were done by the agar-overlay technique. Aliquots of 100 μL of serial 10-fold dilution from the samples were mixed with 100 μL host cells and 4 mL molten LB soft agar (0.6%). The detection limits for bacteria or phage enumeration were 1 CFU/Sample or 10 PFU/Sample in milk samples and 5 CFU/Sample or 50 PFU/Sample in chicken breast and Chinese cabbage samples. Values less than the detection limit for chicken breast and Chinese cabbage samples were replaced with 5 CFU/Sample.

### 2.9. Phage Cocktail Control of SE Mixture

The efficacy of the phage cocktail (combination of PA13076 and PC2184) was studied on food samples (chicken breast, pasteurized whole milk and Chinese cabbage) that were experimentally contaminated with a mixture of equal numbers of *Salmonella* ATCC13076 and CVCC2184. The purified phage stocks of PA13076 and PC2184 were used to make a phage cocktail in SM buffer with a combined titer of 4 × 10^9^ PFU/mL. Briefly, before adding the 25 μL of phage cocktail, the prepared food samples were pre-incubated with 12.5 μL 4 × 10^5^ CFU/ mL of each SE strains and allowed to attach for 15 min. After phages were added, samples were further incubated at 4 °C or 25 °C for up to 5 h. The numbers of viable *Salmonella* concentration were calculated as described above. The detection limits for numbers of bacteria were also the same as above.

### 2.10. Determination of Phage Stability on Foods

Twenty-five microliters of diluted phages (1.6 × 10^10^ PFU/mL) were inoculated directly onto the surface of the meat and cabbage or into the milk sample. These samples were then incubated at 4 °C or 25 °C. The phage titers were determined at 0, 5, 24, 48 and 72 h, separately. The detection limits for phage titers were the same as in Section 2.8.

### 2.11. Statistical Analysis

Bacteria and phage concentrations of each sample were determined by duplicate plating. Results are shown as mean values of the logarithm of CFU/sample and the standard deviations of the mean are indicated by error bars. Each phage-treated sample was compared to its control counterpart using one-way ANOVA. Significant differences were discriminated using Duncan’s test with significance set at *p* < 0.05. All data were analyzed using SPSS 16.0 (SPSS Inc., Chicago, IL, USA).

## 3. Results

### 3.1. Lytic Phages Isolation and Purification

Two new SE phages were isolated and designated as vB_SenM-PA13076 (PA13076) and vB_SenM-PC2184 (PC2184) by their ability to propagate on host strains SE ATCC13076 and CVCC2184, respectively. The bacteria present in the middle of the plaque could not form lysogen. These two phages are indeed strictly lytic. In spot tests, phages PA13076 formed clearing zones on lawns of ATCC13076 and phages PC2184 formed clearing zones on lawns of CVCC2184. Both PC2184 and PA13076 could cross-react and lyse to the other’s hosts. The two phages formed round and clear zones on their own hosts, and the size of plaques were 0.5 to 1 mm.

### 3.2. Phages Morphologies

**Figure 1:**
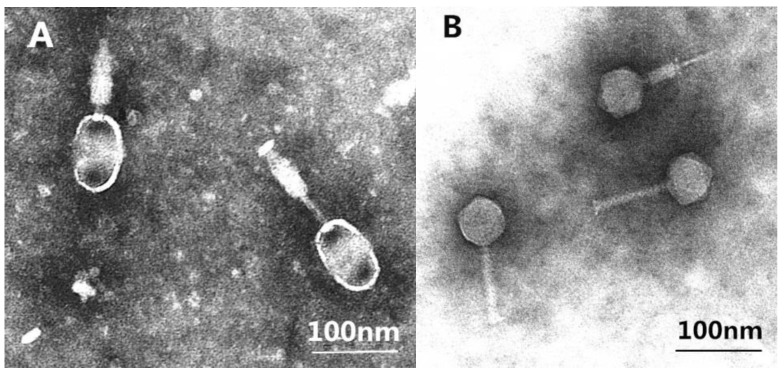
Transmission electron micrographs (TEM) of phage PA13076 (**A**) and PC2184 (**B**).

TEM images of phage PA13076 and PC2184 are shown in Figure 1. They all had the characteristics of a *Myoviridae* family (with a contractile tail). The head of PA13076 was oval and 66 nm in diameter. Its tail was 90 nm in length and 18 nm in diameter. PC2184 possessed an icosahedral head (diameter, 65 nm) and a contractile tail (length, 106 nm and diameter, 17 nm).

### 3.3. Thermal and pH Stability

**Figure 2:**
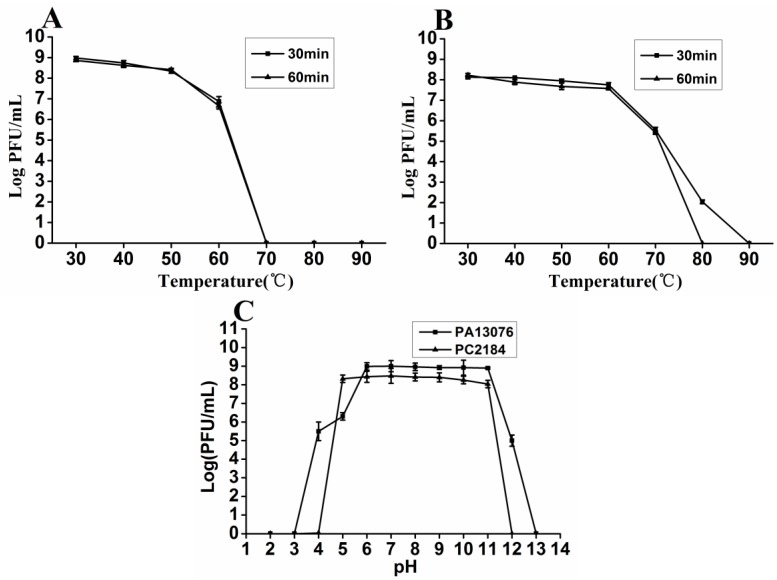
The thermal stability of phages PA13076 (**A**) and PC2184 (**B**); and the pH stability of phages PA13076 and PC2184 (**C**).

Phage PA13076 and PC2184 were stable between 30 °C to 50 °C for 30 min and 60 min. At 60 °C, there was 2-log reduction for PA13076, whereas PC2184 had only a slight reduction. PA13076 were not detectable at 70 °C and PC2184 were not detectable for 60 min at 80 °C (Figure 2A,B). The titers of phage were relatively stable at pH 6 to 11 for PA13076 and at pH 5 to 11 for PC2184. Their titers declined dramatically under lower or higher pH conditions (Figure 2C).

### 3.4. Lytic Activity of Phages PA13076 and PC2184 on Its Host in Vitro

**Figure 3:**
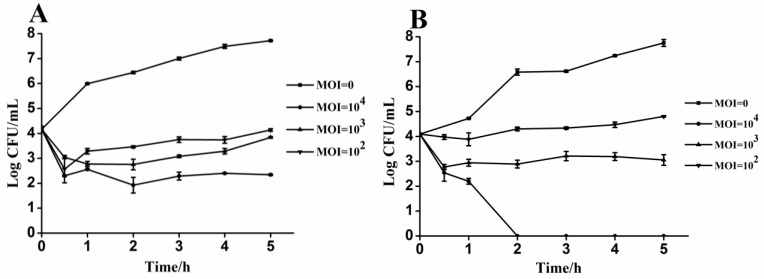
Lytic effects of phage PA13076 and PC2184 against the specified hosts of liquid cultures *in vitro*: (**A**) PA13076 and (**B**) PC2184.

Figure 3A shows the reduction of SE ATCC13076 growth compared to phage-free control (MOI = 0) when phage were added at MOIs of 10^2^, 10^3^ and 10^4^ to host cells initially present at 1.46 × 10^4^ CFU/mL. PA13076 achieved a peak reduction of 0.9, 1.6, and 1.8 log CFU/mL after 0.5 h, respectively. The number of viable SE ATCC13076 was reduced by about 3.5-log when treated with phage at MOI of 10^2^ and 10^3^ compared to the phage-free control after 5 h. Moreover, the number of viable SE ATCC13076 was reduced by 5.5-log when treated with PA13076 at MOI of 10^4^. Figure 3B describes the same experiment using 1.23 × 10^4^ CFU/mL of SE CVCC2184 and different MOIs of PC2184. When the highest MOI (10^4^) of PC2184 was used, SE CVCC2184 numbers were reduced dramatically compared with the control; no viable bacteria were detected by 2 h. The number of viable *Salmonella* remaining at each time was dependent on the MOI used; higher MOI resulted in lower numbers of viable bacteria.

### 3.5. Host Ranges of Phage PA13076 and Phage PC2184

The phage PA13076 and phage PC2184 both possessed wide host ranges. The results indicated that phage PA13076 had a lytic effect on 222 of the 311 epidemic *Salmonella* isolates (71.4%), whereas PC2184 produced a lytic effect on 298 isolates (95.8%) (Supplementary Table S1).

### 3.6. Efficacy of Individual Phage in the Bio-Control SE in Contaminated Foods

**Figure 4:**
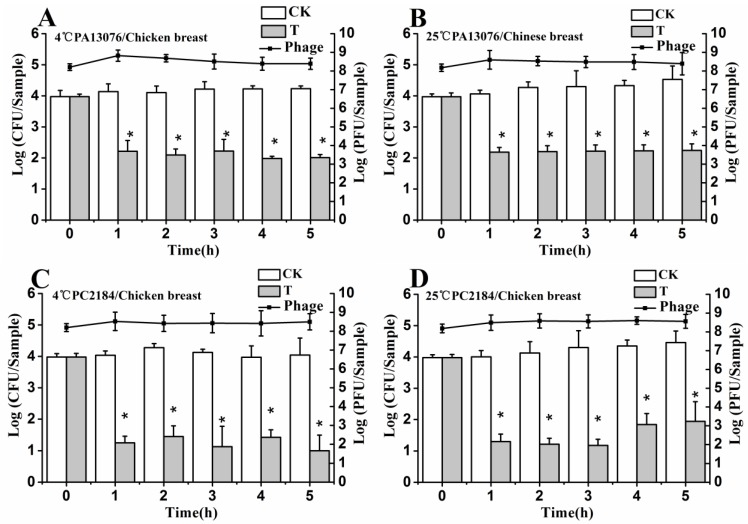
Effects of individual phages on growth of SE ATCC13076 and CVCC2184 on the surface of chicken breast at 4 °C and 25 °C. Each chicken breast sample was inoculated with either SE ATCC13076 (**A**,**B**) or SE CVCC2184 (**C**,**D**) (1 × 10^4^ CFU), and phage PA13076 or PC2184 was applied (1 × 10^8^ PFU) later (CK, inoculated control without phage; T, treated with phage). The titers of phage were also detected each sampling time (indicated in a dotted line). Date represent the mean ± S.D. (*n* = 3), * represents *p* < 0.05 (Duncan’s test).

**Figure 5:**
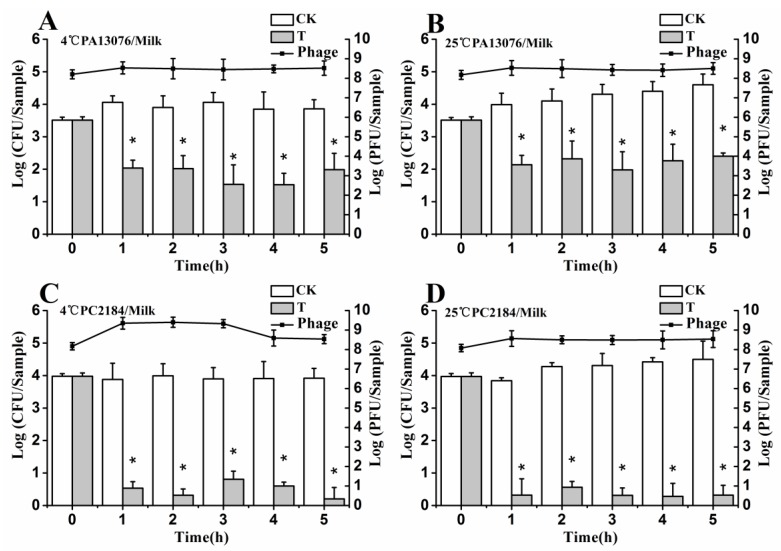
Effects of individual phages on growth of SE ATCC13076 and CVCC2184 in milk samples at 4 °C and 25 °C. Each milk sample was inoculated with either SE ATCC13076 (**A**,**B**) or SE CVCC2184 (**C**,**D**) (1 × 10^4^ CFU), and phage PA13076 or PC2184 was applied (1 × 10^8^ PFU) later (CK, inoculated control without phage; T, treated with phage). The titers of phage were also detected each sampling time (indicated in a dotted line). Date represent the mean ± S.D. (*n* = 3), * represents *p* < 0.05 (Duncan’s test).

**Figure 6:**
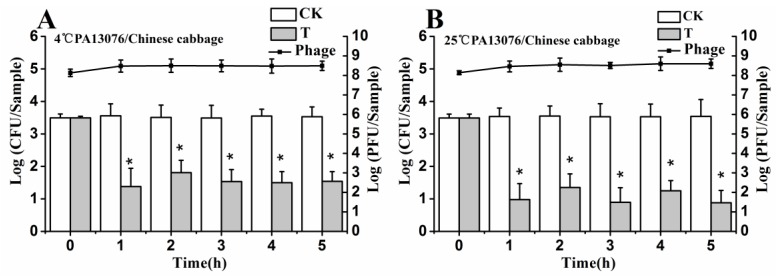

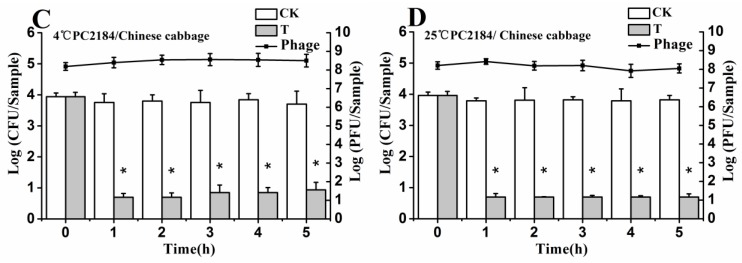
Effects of individual phages on growth of SE ATCC13076 and CVCC2184 on the surface of Chinese cabbage samples at 4 °C and 25 °C. Each sample was inoculated with either SE ATCC13076 (**A**,**B**) or SE CVCC2184 (**C**,**D**) (1 × 10^4^ CFU), and phage PA13076 or PC2184 was applied (1 × 10^8^ PFU) later (CK, inoculated control without phage; T, treated with phage). The titers of phage were also detected each sampling time (indicated in a dotted line). Date represent the mean ± S.D. (*n* = 3), * represents *p* < 0.05 (Duncan’s test).

No host organisms were isolated from uninoculated control samples. The results shown in Figure 4, Figure 5 and Figure 6 demonstrate the effects of single phage PA13076 and phage PC2184 on SE ATCC13076 and SE CVCC2184 contamination, respectively, at 4 and 25 °C on food samples (chicken breast, pasteurized whole milk and Chinese cabbage) and the concentration of phage on the surfaces of these food products at 4 and 25 °C are also shown. The titers of phage PA13076 and PC2184 remained stable and slightly higher than the initial inoculum over 5 h.

The efficacy of phage PC2184 to reduce the number of viable bacteria was clearly better than phage PA13076 at 4 °C and 25 °C. PA13076 and PC2184 treated groups (T) showed significantly lower (*p* < 0.05) SE counts compared with the positive control (CK) group at 4 °C and 25 °C in each of the three foods types. The most significant reductions in viable SE for the two phages were observed in pasteurized whole milk (Figure 5).

### 3.7. Efficacy of Phage Cocktail on Reducing SE Mixture

**Figure 7:**
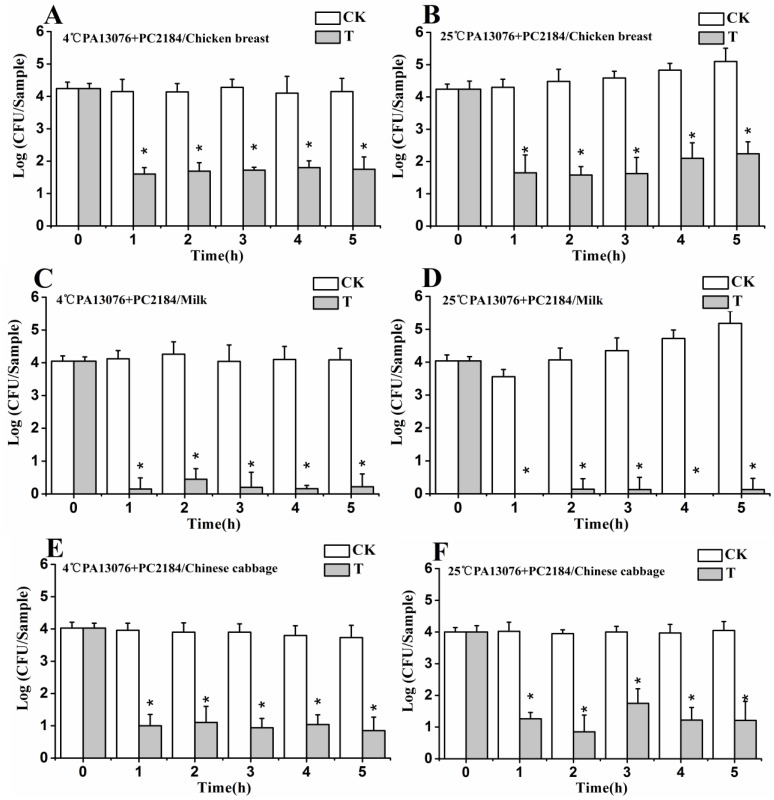
Efficacy of phage cocktail on reducing SE mixture treated food samples at 4 °C and 25 °C: (**A**,**B**) chicken breast; (**C**,**D**) pasteurized whole milk; and (**E**,**F**) Chinese cabbage. Each sample was inoculated with 1 × 10^4^ CFU of SE mix (CK), or 1 × 10^4^ CFU of SE mix and 1 × 10^8^ PFU of phage cocktail (T). Date represent the mean ± S.D. (*n* = 3), * represents *p* < 0.05 (Duncan’s test).

Viable counts increased slightly during incubation of SE mixture (SE ATCC13076 and SE CVCC2184) at 25 °C on chicken breast and in milk, but, at 4 °C they were stable. Bio-control by PA13076 and PC2184 cocktail resulted in a decreasing viable *Salmonella* counts of at least 1.65 log CFU/sample (Figure 7A,B), 3.89 log CFU/sample (Figure 7C,D) and 2.9 log CFU/sample (Figure 7E,F) on the three kinds of foods in the first 1 h, which was followed by a small amount of regrowth during the remaining incubation period at 25 °C. There was almost complete elimination of viable bacteria in pasteurized whole milk at 4 and 25 °C. The effects of the phage cocktail on Chinese cabbage were better than for chicken breast. After 5 h at 4 °C, the SE concentration was reduced by about 3.86 log CFU/sample on Chinese cabbage and 2.5 log CFU/sample on chicken breast relative to the initial concentration of bacteria at 0 h. The reduction in SE counts was somewhat less on Chinese cabbage (3.0 log CFU/sample) at 25 °C after 5 h.

### 3.8. Stability of Phage on the Treated Foods

**Figure 8:**
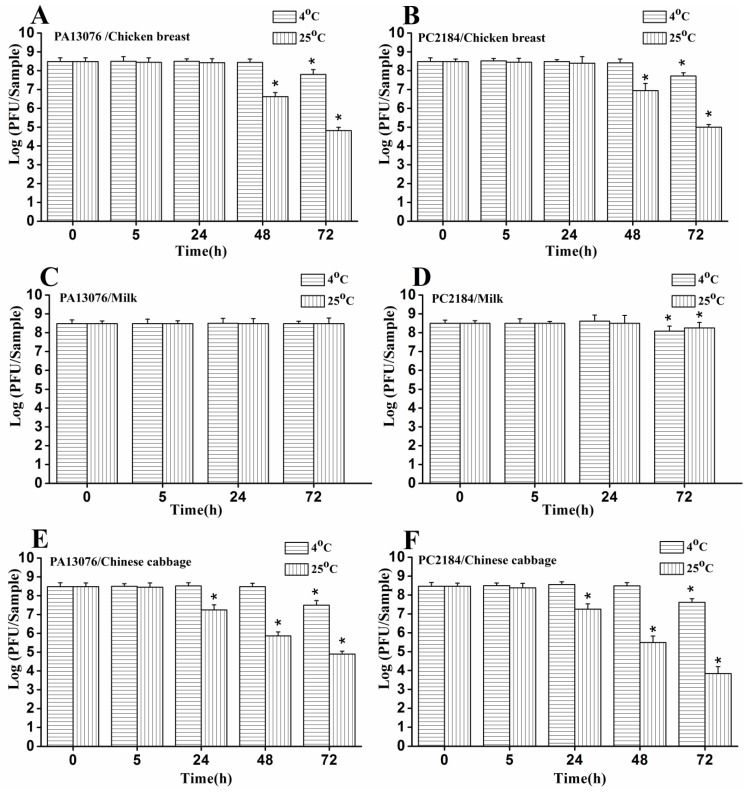
Stability of phage PA13076 and PC2184 over 72 h of incubation on the three kinds of food at 4 °C and 25 °C (the data for the milk sample at 48 h was lacking). Each sample was inoculated with 4 × 10^8^ PFU of phage. Date represent the mean ± S.D. (*n* = 3), * represents *p* < 0.05 compared to the original phage numbers (Duncan’s test).

The concentration of phage PA13076 and phage PC2184 added to the food samples were monitored over 72 h. In milk samples, we observed no significant loss in titers of phage PA13076 at 4 °C and 25 °C (Figure 8C), however there was a significant loss in titers of phage PC2184 at 4 °C and 25 °C at 72 h (Figure 8D). For phage PA13076 and PC2184 added to chicken breast, titers remained stable up to 48 h at 4 °C and up to 24 h at 25 °C (Figure 8A,B). Phage PA13076 and PC2184 all decreased about 2 log CFU/sample and 4 log CFU/sample on chicken breast at 48 and 72 h, respectively, at 25 °C. However, in Chinese cabbage (Figure 8E,F), these two phages are relatively stable up to 48 h at 4 °C and up to 5 h at 25 °C.

## 4. Discussion

In this study, two lytic bacteriophages were successfully isolated from chicken sewages and characterized as *Salmonella* Enteritidis phages vB_SenM-PA13076 (PA13076) and vB_SenM-PC2184 (PC2184) by international common method according to their morphologies and their host’s serovar. PC2184 was very similar to the previously *Salmonella* phage FGCSSa1 in ultrastructure and size [22]. Although, PA13076 belonged to *Myoviridae*, as does PC2184, but the shape of PA13076’s head is very different from other reported *Salmonella* phage *Myoviridae*s [23,24] and *Siphoviruses* [25,26]. Resistance to heat and pH are important for bio-control applications. If these characteristics are too narrow, then phages may be ineffective in actual use. The two phages were both relatively stable between pH 6 and 9, which is compatible with the range (pH 5.5–7.0) of many foods. For thermal tolerance, the two phages were rapidly inactivated if the temperature was higher than 60 °C (for PA13076) or 70 °C (for PC2184), both of the phages were rapidly inactivated. The thermal stability was similar to the *Lactobacillus plantarum* phage PhiJL-1 [27].

As seen in Figure 3, there were differences in the killing ability between the two phages *in vitro*. Phage PC2184 showed impressive killing kinetics in liquid culture, whereas phage PA13076 was not as efficient. In general, the lysis data for the two phages demonstrated that the bactericidal activity was related to the MOI. In the present study, the MOI ratio of 10^4^ showed the highest reduction rate of host cell counts (about 5.5-log for PA13076 and 7.0-log for PC2184). The results demonstrated that phage PC2184 killed its hosts almost completely after 2 h with a sufficiently high MOI (10^4^). Lytic activities of these two phages *in vitro* showed that a much lower ratio of 10^2^ or 10^3^ also gives good killing. Theoretically, a low MOI ratio is advantageous for the commercial feasibility of large-scale application, as it would reduce the cost of preparation, purification and application of phage products. In this assay, we also found that there was significant survival of pathogens when SE cultures were inoculated with phage PA13076 or PC2184 at MOI of 10^2^ and 10^3^. This has previously been shown for the *E. coli* phage Mu^L^ [28] and the *Salmonella* phage FGCSSa1 [22], which did not completely lyse their hosts. Carey-Smith [22] suggested that only a subpopulation of the host was susceptible to phage infection.

Based on the above *in vitro* results, we designed a comprehensive study to determine whether these two phages would significantly reduce the population of *Salmonella* Enteritidis at high MOI (10^4^) grown on foods. Typically, relatively low numbers of *Salmonella* cells are present in foods (including our experiments) [29]. Commercial foods like chicken breast, pasteurized whole milk or Chinese cabbage are usually refrigerated at 4 °C or remain at room temperature (25 °C). Therefore, these two temperatures were chosen to test the activities of individual phages and their combination (cocktail). In the bio-control study, we demonstrated that the individual phage and the cocktail could reduce SE counts on chicken breast surface at 4 °C or 25 °C with a short contact time. In our study, SEs eliminated more counts when the phage cocktail was applied compared to individual phage treatment. Thus, the phage cocktail therapy was more efficacious. These data is consistent with numerous published papers. For example, O’Flynn *et al.*, (2004) confirmed that a phage cocktail with MOI of 10^6^ could completely eliminate *E. coli* O157:H7 in seven of nine cases on meat [30]. Hooton *et al.*, (2011) showed that a mixture of phages (PC1) was more effective at controlling *Salmonella* Typhimurium U288 on pig skin at a MOI of 10 or above [31]. Considering physical limitations of the solid matrix for proper dissemination of phage, applying more phage generally resulted in greater inactivation [32]. Our initial phage doses (10^8^ PFU), which ensure complete contact with *Salmonella* hosts (10^4^ CFU), caused significant reductions of *Salmonella* without the need for phage replicating. These kinds of therapy are called passive, which can reduce the likelihood of development of bacteria resistance [31]. Considering that much lower *Salmonella* concentrations were present, more phage from the beginning of the experiment is necessary to use. Because the growth of SE was greatly suppressed at 4 °C, the ability of phage or phage cocktail gave more favorable results than the experiment at 25 °C.

In experiments on pasteurized whole milk, the ability of the individual phage PC2184 and phage cocktail to control SE was almost indistinguishable. In Guenther’s study [32], similar results were obtained. *Listeria* counts rapidly dropped below detectable levels in liquid foods treated with phages, such as chocolate milk and mozzarella cheese brine, and the levels of residual bacteria were much lower than in other foods [32]. As suggested by Guenther, the greater efficacy of phages in liquid foods may the phage particles being freely suspended. Thus, the individual phage and phage cocktail treatment are likely to be a powerful bio-control method to reduce and possibly eliminate SE in the milk industry.

In recent years, vegetables have been implicated as potential vehicles of bacterial pathogens, including *Salmonella* [33]. In Chinese cabbage bio-control experiment, our results suggested that phage PA13076, PC2184 or the cocktail were each more effective at reducing SE on the surface of Chinese cabbage at 25 °C than at 4 °C, which was opposite to the effects in chicken breast and pasteurized whole milk. This indicates that successful phage bio-control depends on the food matrix and temperature. Previous studies also found that the phage activity was sensitive to the physiological state of the host, which can be affected by growth conditions such as temperature, nutrient availability and oxygen tension [34,35]. These same studies showed that phage ECP-100 application significantly reduced the concentration of viable *E. coli* organisms on tomato slices by *ca*. 99% during storage (10 °C) for 24 h [36].

Phage PA13076 and PC2184 used in this study showed greater stable at 4 °C than at 25 °C. Furthermore, they were more stable in liquid samples (pasteurized whole milk) than in chicken breast and Chinese cabbage; they remained at above 50% of the initial phage concentration. More stable phage have been reported when used in spiced chicken over 72 h at 4 °C [19] and phage P100 in raw salmon fillet tissue over 10 days of storage at 4 °C [37] were reported.

The results obtained in this work have shown that bacteriophage or phage cocktail has the potential to be developed as an alternative strategy to combat SE infection in food. However, it is important to ensure that no horizontal gene transduction occurs while using the phage. Moreover, isolations of new phages having a stronger lytic activity are also needed.

## 5. Conclusions

Our study has shown that bacteriophages or a phage cocktail can reduce or completely eliminate SE inoculated with 10^4^ CFU SE *in vitro* and in foods. These results demonstrated that bacteriophage treatment has the potential to be developed as an alternative strategy to prevent SE infection in food safety. However, more work needs to be done to determine whether phages can be used to disinfect food products.

## Supplementary Files

Supplementary File 1
