# Supplementary material for: Bio-Control of Salmonella Enteritidis in Foods Using Bacteriophages
Source: Viruses. 2015 Aug 24;7(8):4836–53. doi: 10.3390/v7082847 (PMC4576208; doi:10.3390/v7082847)
Supplement: Supplementary File 1 [file viruses-07-02847-s001.pdf]

# Supplementary Materials

**Table S1.** Host ranges of phage PA13076 and phage PC2184.

| No. | *Salmonella* Strains | Serotype | Phage PA13076 Susceptibility [a] | Phage PC2184 Susceptibility [a] | Sources |
|---|---|---|---|---|---|
| 1 | 50336 | Enteritidis | + | +++ | I |
| 2 | 994 | Enteritidis | + | +++ | I |
| 3 | T48 | Enteritidis | ++ | ++ | I |
| 4 | T49 | Enteritidis | ++ | ++ | I |
| 5 | T64 | Enteritidis | + | +++ | I |
| 6 | CVCC1 | Pullorun | − | +++ | I |
| 7 | S11-2 | Pullorun | − | ++ | I |
| 8 | S11-3 | Pullorun | − | +++ | I |
| 9 | S11-4-1 | Pullorun | + | − | I |
| 10 | S11-4-2 | Pullorun | + | +++ | I |
| 11 | S11-5 | Pullorun | + | − | I |
| 12 | S12-2-1 | Pullorun | + | − | I |
| 13 | S11-2-2 | Pullorun | + | − | I |
| 14 | C1 | Enteritidis | + | +++ | II |
| 15 | C2 | Enteritidis | + | +++ | II |
| 16 | C5 | Enteritidis | + | +++ | II |
| 17 | C7 | Enteritidis | + | ++ | II |
| 18 | C8 | Enteritidis | − | ++ | II |
| 19 | C11 | Enteritidis | + | + | II |
| 20 | C12 | Enteritidis | + | +++ | II |
| 21 | C14 | Enteritidis | + | +++ | II |
| 22 | C15 | Enteritidis | + | +++ | II |
| 23 | C16 | Enteritidis | + | +++ | II |
| 24 | C17 | Enteritidis | + | +++ | II |
| 25 | C18 | Enteritidis | + | +++ | II |
| 26 | C19 | Enteritidis | ± | +++ | II |
| 27 | C20 | Enteritidis | ± | +++ | II |
| 28 | C21 | Enteritidis | ± | +++ | II |
| 29 | C22 | Enteritidis | ± | +++ | II |
| 30 | C23 | Enteritidis | ± | +++ | II |
| 31 | C24 | Enteritidis | ± | +++ | II |
| 32 | C25 | Enteritidis | ± | ++ | II |
| 33 | C26 | Enteritidis | − | +++ | II |
| 34 | C27 | Enteritidis | + | +++ | II |
| 35 | C29 | Enteritidis | + | + | II |
| 36 | C30 | Enteritidis | + | +++ | II |
| 37 | C31 | Enteritidis | + | +++ | II |
| 38 | C32 | Enteritidis | + | +++ | II |
| 39 | C36 | Enteritidis | + | +++ | II |
| 40 | C39 | Enteritidis | + | +++ | II |
| 41 | C40 | Enteritidis | + | +++ | II |
| 42 | C41 | Enteritidis | + | +++ | II |

**Table S1.** *Cont.*

| No. | *Salmonella* Strains | Serotype | Phage PA13076 Susceptibility [a] | Phage PC2184 Susceptibility [a] | Sources |
|---|---|---|---|---|---|
| 43 | C43 | Enteritidis | + | +++ | II |
| 44 | C44 | Enteritidis | + | +++ | II |
| 45 | C46 | Enteritidis | + | +++ | II |
| 46 | C53 | Enteritidis | + | +++ | II |
| 47 | C56 | Enteritidis | + | ++ | II |
| 48 | C57 | Enteritidis | + | +++ | II |
| 49 | C58 | Enteritidis | − | +++ | II |
| 50 | C59 | Enteritidis | − | +++ | II |
| 51 | C60 | Enteritidis | − | +++ | II |
| 52 | C61 | Enteritidis | − | +++ | II |
| 53 | C63 | Enteritidis | + | +++ | II |
| 54 | C65 | Enteritidis | + | +++ | II |
| 55 | C71 | Enteritidis | + | ++ | II |
| 56 | C72 | Enteritidis | + | +++ | II |
| 57 | C76 | Enteritidis | + | ++ | II |
| 58 | C79 | Enteritidis | + | +++ | II |
| 59 | C80 | Enteritidis | + | +++ | II |
| 60 | C83 | Enteritidis | + | ++ | II |
| 61 | C88 | Enteritidis | + | ++ | II |
| 62 | C89 | Enteritidis | + | ++ | II |
| 63 | C94 | Enteritidis | + | ++ | II |
| 64 | C96 | Enteritidis | + | ++ | II |
| 65 | C99 | Enteritidis | + | + | II |
| 66 | C102 | Enteritidis | + | +++ | II |
| 67 | C105 | Enteritidis | + | ++ | II |
| 68 | C106 | Enteritidis | + | ++ | II |
| 69 | C110 | Enteritidis | + | ++ | II |
| 70 | C118 | Enteritidis | + | ++ | II |
| 71 | C124 | Enteritidis | + | ++ | II |
| 72 | C125 | Enteritidis | + | ++ | II |
| 73 | C128 | Enteritidis | + | ++ | II |
| 74 | C130 | Enteritidis | + | + | II |
| 75 | C133 | Enteritidis | − | ++ | II |
| 76 | C135 | Enteritidis | + | + | II |
| 77 | C137 | Enteritidis | − | + | II |
| 78 | C138 | Enteritidis | + | ++ | II |
| 79 | C139 | Enteritidis | + | ++ | II |
| 80 | C144 | Enteritidis | + | ++ | II |
| 81 | C145 | Enteritidis | + | + | II |
| 82 | C146 | Enteritidis | + | + | II |
| 83 | C149 | Enteritidis | + | ++ | II |
| 84 | C150 | Enteritidis | + | + | II |
| 85 | C152 | Enteritidis | + | + | II |

**Table S1.** *Cont.*

| No. | *Salmonella* Strains | Serotype | Phage PA13076 Susceptibility [a] | Phage PC2184 Susceptibility [a] | Sources |
|---|---|---|---|---|---|
| 86 | C154 | Enteritidis | + | +++ | II |
| 87 | C155 | Enteritidis | + | + | II |
| 88 | C156 | Enteritidis | + | ++ | II |
| 89 | C158 | Enteritidis | + | +++ | II |
| 90 | C161 | Enteritidis | + | ++ | II |
| 91 | C164 | Enteritidis | + | ++ | II |
| 92 | C165 | Enteritidis | + | ++ | II |
| 93 | C167 | Enteritidis | + | ++ | II |
| 94 | 2 | Enteritidis | − | − | II |
| 95 | 4 | Enteritidis | − | − | II |
| 96 | 8 | Enteritidis | + | + | II |
| 97 | 21 | Enteritidis | + | +++ | II |
| 98 | 31 | Enteritidis | + | + | II |
| 99 | 32 | Enteritidis | + | + | II |
| 100 | 34 | Enteritidis | + | + | II |
| 101 | 36 | Enteritidis | + | + | II |
| 102 | 37 | Enteritidis | + | + | II |
| 103 | 42 | Enteritidis | + | +++ | II |
| 104 | 43 | Enteritidis | − | ++ | II |
| 105 | 59 | Enteritidis | + | + | II |
| 106 | 71 | Enteritidis | + | + | II |
| 107 | SM-1-KDE | Enteritidis | + | ++ | II |
| 108 | SM-3-KDE | Indiana | + | + | II |
| 109 | SM-4-KDE | Typhimurium | − | ++ | II |
| 110 | SM-5-KDE | Typhimurium | − | ++ | II |
| 111 | SM-6-KDE | Typhimurium | − | ++ | II |
| 112 | SM-7-KDE | Typhimurium | − | ++ | II |
| 113 | SM-8-KDE | Typhimurium | + | ++ | II |
| 114 | SM-10-KDE | Enteritidis | + | ++ | II |
| 115 | SM-12-KDE | Typhimurium | − | ++ | II |
| 116 | SM-14-KDE | Enteritidis | + | ++ | II |
| 117 | SM-16-KDE | Enteritidis | + | +++ | II |
| 118 | SM-17-KDE | Enteritidis | + | +++ | II |
| 119 | SM-22-KDE | Newport | + | + | II |
| 120 | SM-23-KDE | Enteritidis | + | ++ | II |
| 121 | SM-27-KDE | Sanftenberg | + | + | II |
| 122 | SM-28-KDE | Indiana | + | ++ | II |
| 123 | SM-29-KDE | Kottbus | + | ++ | II |
| 124 | SM-30-KDE | Indiana | + | ++ | II |
| 125 | SM-31-KDE | Choleraesuis | + | + | II |
| 126 | SM-32-KDE | Enteritidis | + | ++ | II |
| 127 | SM-34-KDE | Enteritidis | + | +++ | II |
| 128 | SM-38-KDE | Enteritidis | + | +++ | II |
| 129 | SM-39-KDE | Enteritidis | ++ | − | II |

**Table S1.** *Cont.*

| No. | *Salmonella* Strains | Serotype | Phage PA13076 Susceptibility [a] | Phage PC2184 Susceptibility [a] | Sources |
|---|---|---|---|---|---|
| 130 | SM-41-KDE | Enteritidis | − | ++ | II |
| 131 | SM-46-KDE | Enteritidis | + | +++ | II |
| 132 | SM-49-KDE | Enteritidis | + | − | II |
| 133 | SM-52-KDE | Enteritidis | + | ++ | II |
| 134 | SM-53-KDE | Enteritidis | + | +++ | II |
| 135 | SM-54-KDE | Enteritidis | + | +++ | II |
| 136 | SM-55-KDE | Enteritidis | + | +++ | II |
| 137 | SM-56-KDE | Indiana | + | ++ | II |
| 138 | SM-57-KDE | Indiana | + | +++ | II |
| 139 | SM-58-KDE | Typhimurium | − | + | II |
| 140 | SM-61-KDE | Newport | ++ | +++ | II |
| 141 | SM-SC-XT-1 | Enteritidis | − | +++ | II |
| 142 | SM-SC-XT-2 | Enteritidis | − | +++ | II |
| 143 | SM-SC-XT-3 | Enteritidis | + | +++ | II |
| 144 | SM-SC-XT-4 | Enteritidis | + | +++ | II |
| 145 | SM-SC-XT-5 | Enteritidis | − | +++ | II |
| 146 | SM-SC-XT-6 | Enteritidis | − | +++ | II |
| 147 | SM-SC-XT-7 | Enteritidis | + | ++ | II |
| 148 | SM-SC-XT-8 | Enteritidis | + | +++ | II |
| 149 | SM-SC-XT-9 | Enteritidis | + | +++ | II |
| 150 | SM-SC-XT-10 | Enteritidis | + | +++ | II |
| 151 | SM-SC-XT-11 | Enteritidis | + | +++ | II |
| 152 | SM-SC-XT-12 | Enteritidis | − | +++ | II |
| 153 | SM-SC-XT-13 | Enteritidis | + | +++ | II |
| 154 | SM-SC-XT-14 | Enteritidis | − | +++ | II |
| 155 | SM-SC-XT-15 | Enteritidis | − | +++ | II |
| 156 | SM-SC-XT-16 | Enteritidis | − | +++ | II |
| 157 | SM-SC-XT-17 | Enteritidis | − | +++ | II |
| 158 | SM-SC-XT-18 | Enteritidis | − | +++ | II |
| 159 | SM-SC-XT-19 | Enteritidis | + | +++ | II |
| 160 | SM-SC-XT-20 | Enteritidis | − | +++ | II |
| 161 | SM-SC-XT-21 | Enteritidis | − | ++ | II |
| 162 | SM-SC-XT-22 | Enteritidis | − | ++ | II |
| 163 | SM-SC-XT-23 | Enteritidis | − | ++ | II |
| 164 | SM-SC-XT-24 | Enteritidis | − | ++ | II |
| 165 | SM-SC-XT-25 | Enteritidis | − | +++ | II |
| 166 | SM-SC-XT-26 | Enteritidis | − | ++ | II |
| 167 | SM-SC-XT-27 | Enteritidis | + | ++ | II |
| 168 | SM-SC-XT-28 | Enteritidis | + | ++ | II |
| 169 | SM-SC-XT-29 | Enteritidis | + | ++ | II |
| 170 | SM-SC-XT-30 | Enteritidis | − | ++ | II |
| 171 | SM-SC-XT-31 | Enteritidis | − | + | II |
| 172 | SM-SC-XT-32 | Enteritidis | + | +++ | II |
| 173 | SM-SC-XT-33 | Enteritidis | + | +++ | II |

**Table S1.** *Cont.*

| No. | *Salmonella* Strains | Serotype | Phage PA13076 Susceptibility [a] | Phage PC2184 Susceptibility [a] | Sources |
|---|---|---|---|---|---|
| 174 | SM-SC-XT-34 | Enteritidis | + | +++ | II |
| 175 | SM-SC-XT-35 | Enteritidis | − | +++ | II |
| 176 | SM-SC-XT-36 | Enteritidis | + | ++ | II |
| 177 | SM-SC-XT-37 | Enteritidis | + | ++ | II |
| 178 | SM-SC-XT-38 | Enteritidis | + | ++ | II |
| 179 | SM-90-1 | Enteritidis | − | +++ | II |
| 180 | SM-90-2 | Enteritidis | − | +++ | II |
| 181 | SC-81 | Enteritidis | − | − | II |
| 182 | SC-91 | Typhimurium | ++ | − | II |
| 183 | SC-131 | Enteritidis | − | ++ | II |
| 184 | L3 | Enteritidis | − | +++ | II |
| 185 | L5 | Enteritidis | + | +++ | II |
| 186 | L6 | Enteritidis | + | +++ | II |
| 187 | L7 | Enteritidis | − | +++ | II |
| 188 | L10 | Enteritidis | − | + | II |
| 189 | L11 | Enteritidis | − | ++ | II |
| 190 | L13 | Enteritidis | + | +++ | II |
| 191 | L20 | Enteritidis | − | + | II |
| 192 | L24 | Enteritidis | − | +++ | II |
| 193 | L35 | Enteritidis | + | +++ | II |
| 194 | L40 | Enteritidis | + | ++ | II |
| 195 | L43 | Enteritidis | − | ++ | II |
| 196 | L44 | Enteritidis | + | ++ | II |
| 197 | L56 | Derby | − | − | II |
| 198 | L80 | Indiana | + | + | II |
| 199 | L98 | Indiana | + | + | II |
| 200 | L333 | Enteritidis | +++ | − | II |
| 201 | 205 | Enteritidis | + | ++ | II |
| 202 | 206 | Enteritidis | + | ++ | II |
| 203 | 213 | Enteritidis | − | +++ | II |
| 204 | 225 | Indiana | + | ++ | II |
| 205 | 228 | Enteritidis | − | ++ | II |
| 206 | 230 | Indiana | + | ++ | II |
| 207 | 234 | Enteritidis | − | ++ | II |
| 208 | 243 | Indiana | − | +++ | II |
| 209 | 269 | Enteritidis | + | +++ | II |
| 210 | 275 | not belong A-f group | ++ | − | II |
| 211 | 283 | Indiana | + | ++ | II |
| 212 | 300 | Indiana | + | ++ | II |
| 213 | 307 | Indiana | + | ++ | II |
| 214 | 312 | Enteritidis | − | ++ | II |
| 215 | 315 | Indiana | + | ++ | II |
| 216 | 324 | Enteritidis | − | +++ | II |
| 217 | 325 | Enteritidis | − | ++ | II |

**Table S1.** *Cont.*

| No. | *Salmonella* Strains | Serotype | Phage PA13076 Susceptibility [a] | Phage PC2184 Susceptibility [a] | Sources |
|---|---|---|---|---|---|
| 218 | 334 | Enteritidis | + | +++ | II |
| 219 | 342 | Indiana | + | ++ | II |
| 220 | 343 | Indiana | + | ++ | II |
| 221 | 354 | Enteritidis | + | ++ | II |
| 222 | 359 | Indiana | + | +++ | II |
| 223 | 360 | Enteritidis | + | +++ | II |
| 224 | 365 | Enteritidis | − | +++ | II |
| 225 | 404 | Indiana | + | +++ | II |
| 226 | 407 | Typhimurium | − | − | II |
| 227 | 410 | Enteritidis | + | +++ | II |
| 228 | 414 | Indiana | + | +++ | II |
| 229 | 415 | Indiana | + | +++ | II |
| 230 | 418 | Enteritidis | + | +++ | II |
| 231 | 420 | Indiana | + | ++ | II |
| 232 | 421 | Enteritidis | + | +++ | II |
| 233 | 431 | Enteritidis | − | ++ | II |
| 234 | 433 | Enteritidis | + | +++ | II |
| 235 | 450 | Enteritidis | + | +++ | II |
| 236 | 457 | Enteritidis | − | +++ | II |
| 237 | 460 | Indiana | + | +++ | II |
| 238 | 462 | Indiana | + | +++ | II |
| 239 | 470 | Enteritidis | + | +++ | II |
| 240 | 472 | Enteritidis | + | +++ | II |
| 241 | 505 | Enteritidis | + | ++ | II |
| 242 | 507 | Enteritidis | + | +++ | II |
| 243 | 508 | Enteritidis | − | +++ | II |
| 244 | 513 | Enteritidis | + | +++ | II |
| 245 | 515 | Enteritidis | + | +++ | II |
| 246 | 517 | Enteritidis | ++ | ++ | II |
| 247 | 521 | Typhimurium | + | ++ | II |
| 248 | SPu-109 | Pullorum | − | + | III |
| 249 | SPu-13 | Pullorum | − | − | III |
| 250 | SPu-102 | Pullorum | − | − | III |
| 251 | SPu-45 | Pullorum | − | + | III |
| 252 | SPu-115 | Pullorum | − | + | III |
| 253 | SPu-116 | Pullorum | − | +++ | III |
| 254 | SPu-905 | Pullorum | − | + | III |
| 255 | SPu-27 | Pullorum | − | − | III |
| 256 | SPu-01 | Pullorum | − | + | III |
| 257 | SPu-49 | Pullorum | − | + | III |
| 258 | SPu-103 | Pullorum | − | − | III |
| 259 | SPu-85 | Pullorum | − | + | III |
| 260 | E2 | Typhimurium | ++ | ++ | III |
| 261 | E6 | Typhimurium | + | − | III |

**Table S1.** *Cont.*

| No. | *Salmonella* Strains | Serotype | Phage PA13076 Susceptibility [a] | Phage PC2184 Susceptibility [a] | Sources |
|---|---|---|---|---|---|
| 262 | E10 | Typhimurium | + | ++ | III |
| 263 | E12 | Typhimurium | + | ++ | III |
| 264 | E16 | Typhimurium | + | ++ | III |
| 265 | E17 | Anatum | + | + | III |
| 266 | D9 | London | + | +++ | III |
| 267 | S3 | Typhimurium | + | ++ | III |
| 268 | S8 | Typhimurium | + | ++ | III |
| 269 | S9 | Typhimurium | + | ++ | III |
| 270 | S20 | Enteritidis | + | ++ | III |
| 271 | DHLM-1 | Typhimurium | + | + | III |
| 272 | DHLM-2 | Typhimurium | + | + | III |
| 273 | JKSen | Enteritidis | + | ++ | III |
| 274 | C50041 | Enteritidis | − | − | III |
| 275 | CYR1 | Enteritidis | +++ | +++ | III |
| 276 | CYR2 | Enteritidis | + | ++ | III |
| 277 | CYR3 | Enteritidis | + | +++ | III |
| 278 | CYR4 | Enteritidis | + | + | III |
| 279 | CYR5 | Enteritidis | + | + | III |
| 280 | ATCC13076 | Enteritidis | +++ | +++ | IV |
| 281 | ATCC13311 | Typhimurium | − | +++ | IV |
| 282 | ATCC50073 | Paratyphi A | − | +++ | IV |
| 283 | CVCC2184 | Enteritidis | + | +++ | III |
| 284 | CMCC533 | Pullorum | − | ++ | III |
| 285 | HNER027 | Derby | + | + | V |
| 286 | HNER121 | Enteritidis | + | ++ | V |
| 287 | HNER055 | Derby | + | + | V |
| 288 | HNER127 | Derby | + | ++ | V |
| 289 | HNER004 | Derby | + | ++ | V |
| 290 | HNER067-1 | Derby | ++ | ++ | V |
| 291 | HNER067-2 | Derby | + | +++ | V |
| 292 | HNER117 | Derby | ++ | +++ | V |
| 293 | HNER102 | Derby | + | +++ | V |
| 294 | HNER047 | Derby | + | + | V |
| 295 | HNER178 | Typhimurium | + | ++ | V |
| 296 | HNER086 | Derby | ++ | +++ | V |
| 297 | CDER157 | O:10, H:1,w | − | ++ | V |
| 298 | CDER160 | Derby | + | ++ | V |
| 299 | CDER188 | Derby | + | +++ | V |
| 300 | DBER023 | Typhimurium | + | ++ | V |
| 301 | DLJR01 | Arizonae | − | − | V |
| 302 | DBJR151 | Enteritidis | + | ++ | V |
| 303 | DBJR193-2 | Derby | + | ++ | V |
| 304 | DBJR236 | Enteritidis | + | + | V |
| 305 | 1769 | Derby | + | − | VI |

**Table S1.** *Cont.*

| No. | *Salmonella* Strains | Serotype | Phage PA13076 Susceptibility [a] | Phage PC2184 Susceptibility [a] | Sources |
|---|---|---|---|---|---|
| 306 | 1401 | Pullorum | − | + | VI |
| 307 | 1402 | Pullorum | − | − | VI |
| 308 | 1403 | Pullorum | − | − | VI |
| 309 | 1404 | Pullorum | − | + | VI |
| 310 | 1405 | Pullorum | − | +++ | VI |
| 311 | 1406 | Pullorum | − | ++ | VI |

Sources: I Guo-qiang Zhu (Yangzhou University, Yangzhou, China); II Yu-qing Liu (Shandong Academy of Agricultural Sciences, Jinan, China ); III Lab stock; IV Guo-xiang Cao (Chinese Academy of Aricultural Science, Yangzhou, China); V Yan-bin Zeng (Jiangxi Academy of Agricultural Sciences, Nanchang, China); VI Jian-sen Gong(Poultry institute, Chinese Academy of Aricultural Sciences, Yangzhou, China, Yangzhou, China); [a] +++, complete lysis; ++, lysis; +, turbid lysis; −, no plagues.
